# Supplementary material for: A Likelihood Approach for Real-Time Calibration of Stochastic Compartmental Epidemic Models
Source: PLoS Comput Biol. 2017 Jan 17;13(1):e1005257. doi: 10.1371/journal.pcbi.1005257 (PMC5240920; doi:10.1371/journal.pcbi.1005257)
Supplement: S1 File — (TAR.GZ) [file pcbi.1005257.s014.tar.gz › HSPH_Online-SI-Revision/IPoi09/n10-extreme/n10-extreme_BENCH_table-4.pdf]

| | | | | | | | | | | | | | | | | | | | | | | | | | | | |
|---|---|---|---|---|---|---|---|---|---|---|---|---|---|---|---|---|---|---|---|---|---|---|---|---|---|---|---|
| 1 | R0 | {2.68692, 12.5884} | {2.62463, 12.4381} | {{2.32678, 10.0266}, {2.81617, 13.9425}} | {5.81896, 9.05574} | inf | 652. | 1155.81 | {443., 1905.} | 95.4974 | 0.313621 | newly | 547. | 821.829 | {330., 1288.} | 70.0862 | 0.268235 | Reff | 2.38787 | 2.07739 | {1.55945, 2.53694} | 16.6165 | ar | 9089. | 9053.74 | {8715., 9283.} | 1.99393 |
| 2 | R0 | {1.79802, 12.5412} | {1.95511, 10.451} | {{1.85533, 9.28761}, {2.0968, 12.1899}} | {8.99302, 17.9753} | inf | 658. | 1017.03 | {370., 1509.} | 75.2786 | 0.278603 | newly | 408. | 730.939 | {309., 989.} | 92.7733 | 0.305195 | Reff | 1.51609 | 1.37882 | {0.961547, 1.83427} | 20.2712 | ar | 7412. | 7801.33 | {7443., 8277.} | 5.62969 |
| 3 | R0 | {2.6174, 10.1105} | {2.79439, 10.3767} | {{2.59708, 9.39342}, {2.98791, 11.317}} | {8.37559, 7.24116} | inf | 487. | 566.07 | {157., 1035.} | 59.5135 | 0.277388 | newly | 540. | 608.944 | {183., 1014.} | 51.6927 | 0.246593 | Reff | 2.41377 | 2.52953 | {2.3059, 2.77798} | 8.22654 | ar | 9109. | 9222.3 | {9049., 9390.} | 1.99988 |
| 4 | R0 | {2.27532, 14.3149} | {2.8435, 18.1831} | {{2.5458, 15.1849}, {2.97157, 19.3547}} | {24.9714, 27.0224} | inf | 1121. | 1664.54 | {692., 2537.} | 68.5953 | 0.266297 | newly | 626. | 736.985 | {341., 1037.} | 40.9634 | 0.20117 | Reff | 1.73038 | 1.99143 | {1.42843, 2.60399} | 24.31 | ar | 8478. | 9276.23 | {9019., 9420.} | 9.41532 |
| 5 | R0 | {2.96649, 18.0674} | {2.4351, 10.9266} | {{2.22376, 9.35432}, {2.7943, 13.5962}} | {17.913, 39.5232} | inf | 1477. | 2031.22 | {1540., 2533.} | 42.5392 | 0.161308 | newly | 689. | 1248.9 | {987., 1424.} | 84.5691 | 0.271205 | Reff | 2.25186 | 1.07556 | {0.661454, 1.63939} | 52.625 | ar | 9476. | 8831.86 | {8398., 9241.} | 6.79765 |
| 6 | R0 | {2.94778, 16.0002} | {1.96902, 7.1837} | {{1.80312, 5.98216}, {2.36298, 10.1198}} | {33.2033, 55.1024} | inf | 820. | 638.258 | {93., 1225.} | 48.7569 | 0.372328 | newly | 489. | 757.468 | {130., 1332.} | 85.4245 | 0.319662 | Reff | 2.5348 | 1.68488 | {1.28112, 2.19401} | 33.5301 | ar | 9321. | 7783.63 | {7173., 8881.} | 16.4936 |
| 7 | R0 | {2.17562, 11.7184} | {2.14382, 7.66165} | {{1.83544, 5.78475}, {2.68409, 10.6354}} | {14.2138, 35.7909} | inf | 569. | 961.016 | {259., 1776.} | 96.1113 | 0.306247 | newly | 469. | 1044.25 | {370., 1557.} | 132.856 | 0.345228 | Reff | 1.95066 | 1.70878 | {1.2191, 2.24903} | 21.5422 | ar | 8345. | 8222.32 | {7383., 9197.} | 8.23704 |
| 8 | R0 | {2.7904, 17.8966} | {2.83155, 16.5574} | {{2.58655, 14.3523}, {2.96398, 17.7348}} | {3.98317, 7.67275} | inf | 1183. | 2085.09 | {805., 2879.} | 87.9876 | 0.279549 | newly | 589. | 963.173 | {493., 1199.} | 72.5501 | 0.241707 | Reff | 2.25715 | 1.67214 | {1.03071, 2.28329} | 28.5806 | ar | 9285. | 9268.13 | {9018., 9411.} | 1.17307 |
| 9 | R0 | {2.51574, 16.376} | {2.56627, 13.526} | {{2.28112, 11.2541}, {2.7499, 15.3124}} | {6.59131, 17.4037} | inf | 1086. | 1662.13 | {771., 2524.} | 68.5665 | 0.264052 | newly | 556. | 966.529 | {601., 1262.} | 83.9346 | 0.295687 | Reff | 2.01712 | 1.66797 | {1.0557, 2.20035} | 22.5884 | ar | 8937. | 8966.81 | {8537., 9214.} | 2.49146 |
| 10 | R0 | {2.56405, 18.1787} | {2.16341, 10.5295} | {{1.93419, 8.56054}, {2.43464, 12.8735}} | {16.3619, 42.0777} | inf | 985. | 1563.23 | {927., 2132.} | 64.8017 | 0.210935 | newly | 476. | 1014.73 | {807., 1214.} | 115.366 | 0.329283 | Reff | 2.09996 | 1.22364 | {0.775417, 1.77406} | 42.1592 | ar | 9005. | 8383.77 | {7744., 8992.} | 7.24892 |
| 11 | R0 | {2.475, 17.1709} | {2.42338, 19.4921} | {{2.42625, 19.5287}, {2.42625, 19.5287}} | {2.08563, 13.6318} | inf | 1114. | 1269.9 | {122., 2203.} | 58.1533 | 0.339832 | newly | 539. | 495.069 | {62., 804.} | 41.8783 | 0.309593 | Reff | 1.97134 | 1.77387 | {1.1697, 2.37239} | 19.1253 | ar | 8870. | 8818.41 | {8741., 8904.} | 0.765074 |
| 12 | R0 | {2.14974, 11.8619} | {2.19284, 11.9079} | {{1.75445, 7.55154}, {2.75274, 17.3228}} | {13.6243, 23.7212} | inf | 514. | 644.419 | {120., 1148.} | 59.464 | 0.258727 | newly | 376. | 475.387 | {140., 857.} | 57.9444 | 0.243473 | Reff | 1.93176 | 1.93345 | {1.59592, 2.43461} | 15.6366 | ar | 8414. | 8288.95 | {7196., 9262.} | 7.22778 |
| 13 | R0 | {2.20385, 14.4767} | {2.64373, 19.7855} | {{2.64373, 19.7855}, {2.64373, 19.7855}} | {19.9596, 36.6713} | inf | 1178. | 1251.79 | {228., 2250.} | 52.35 | 0.304143 | newly | 681. | 510.921 | {112., 865.} | 36.2189 | 0.283951 | Reff | 1.66898 | 2.03333 | {1.50825, 2.53269} | 25.8759 | ar | 8347. | 9088.49 | {9032., 9142.} | 8.88334 |
| 14 | R0 | {2.35671, 13.8357} | {2.46222, 11.0471} | {{2.22376, 9.35432}, {2.70836, 12.8589}} | {9.30071, 20.2256} | inf | 1020. | 1699.57 | {847., 2486.} | 76.786 | 0.25316 | newly | 683. | 1189.29 | {792., 1446.} | 81.4268 | 0.266492 | Reff | 1.89291 | 1.53491 | {0.942829, 2.18328} | 25.3629 | ar | 8719. | 8835.07 | {8341., 9207.} | 3.98879 |
| 15 | R0 | {1.91368, 10.1082} | {2.82551, 18.7684} | {{2.68803, 17.202}, {2.93978, 19.9817}} | {47.6481, 85.6753} | inf | 746. | 1965.9 | {1041., 2764.} | 167.854 | 0.416477 | newly | 600. | 809.588 | {490., 1036.} | 45.9657 | 0.180983 | Reff | 1.56616 | 1.76365 | {1.13489, 2.38593} | 26.0185 | ar | 7486. | 9267.65 | {9138., 9388.} | 23.7997 |
| 16 | R0 | {2.48727, 14.882} | {2.55131, 13.2181} | {{2.28112, 11.1899}, {2.76203, 15.3124}} | {7.23837, 12.5052} | inf | 818. | 711.347 | {192., 1334.} | 47.0473 | 0.300931 | newly | 517. | 508.083 | {148., 955.} | 46.4002 | 0.26522 | Reff | 2.15174 | 2.20886 | {1.87762, 2.54659} | 11.5661 | ar | 8894. | 8934.94 | {8649., 9216.} | 2.30243 |
| 17 | R0 | {2.46795, 13.1915} | {2.2853, 8.37408} | {{2.05986, 6.93093}, {2.68409, 10.6354}} | {11.4484, 36.5191} | inf | 685. | 688.238 | {100., 1349.} | 55.7572 | 0.349879 | newly | 499. | 786.825 | {149., 1443.} | 87.6897 | 0.343485 | Reff | 2.17698 | 1.98619 | {1.54943, 2.37116} | 14.0124 | ar | 8796. | 8520.4 | {8006., 9117.} | 5.04288 |
| 18 | R0 | {2.96603, 16.7543} | {2.64555, 13.1312} | {{2.44675, 11.6528}, {2.91192, 15.1048}} | {10.8272, 21.6249} | inf | 1177. | 1060.18 | {380., 2008.} | 43.6154 | 0.254634 | newly | 657. | 710.088 | {269., 1180.} | 43.9699 | 0.216667 | Reff | 2.40011 | 2.16675 | {1.63092, 2.64528} | 14.568 | ar | 9394. | 9096.93 | {8792., 9383.} | 3.18241 |
| 19 | R0 | {2.25216, 9.90465} | {2.6538, 12.1246} | {{2.33405, 10.0266}, {2.96307, 14.3219}} | {18.2509, 23.5429} | inf | 358. | 468.075 | {95., 923.} | 69.8199 | 0.29271 | newly | 390. | 402.97 | {85., 768.} | 50.3674 | 0.269204 | Reff | 2.11343 | 2.4571 | {2.091, 2.76563} | 17.6335 | ar | 8631. | 9089.07 | {8685., 9361.} | 5.77734 |
| 20 | R0 | {2.70837, 18.1923} | {2.80833, 16.4573} | {{2.52651, 14.1724}, {2.98558, 18.1089}} | {6.50422, 9.53725} | inf | 1099. | 945.306 | {338., 1706.} | 40.7188 | 0.230736 | newly | 555. | 505.611 | {214., 843.} | 35.3153 | 0.188752 | Reff | 2.21924 | 2.37225 | {2.01852, 2.66763} | 11.0956 | ar | 9183. | 9259.33 | {9037., 9415.} | 1.64344 |
| 21 | R0 | {2.30288, 15.5877} | {2.61932, 18.4036} | {{2.53519, 17.5902}, {2.76795, 19.6041}} | {13.741, 18.0646} | inf | 1079. | 1607.77 | {573., 2418.} | 71.418 | 0.297337 | newly | 546. | 672.967 | {273., 949.} | 47.6252 | 0.239895 | Reff | 1.82273 | 1.79739 | {1.17887, 2.40353} | 20.54 | ar | 8674. | 9077.9 | {8977., 9228.} | 4.65644 |
| 22 | R0 | {2.82208, 10.7544} | {2.46037, 9.50564} | {{1.89934, 6.19362}, {2.96796, 12.6436}} | {14.8135, 18.516} | inf | 295. | 331.857 | {40., 664.} | 66.2349 | 0.366879 | newly | 339. | 386.357 | {48., 768.} | 63.5904 | 0.353109 | Reff | 2.6838 | 2.36873 | {1.95229, 2.87031} | 14.636 | ar | 9333. | 8822.66 | {7883., 9386.} | 5.72833 |
| 23 | R0 | {1.69923, 10.1366} | {1.90362, 8.5074} | {{1.89254, 8.41615}, {1.95355, 8.88568}} | {12.0286, 16.4206} | inf | 645. | 1192.04 | {800., 1475.} | 89.8201 | 0.277007 | newly | 502. | 975.679 | {682., 1166.} | 97.8388 | 0.294045 | Reff | 1.4056 | 1.11612 | {0.785972, 1.48278} | 24.865 | ar | 7055. | 7703.74 | {7539., 7920.} | 9.31094 |
| 24 | R0 | {2.64944, 19.054} | {2.9061, 19.6029} | {{2.76849, 18.33}, {2.93978, 19.9817}} | {9.6875, 4.5197} | inf | 1801. | 2265.69 | {901., 2945.} | 44.6254 | 0.199184 | newly | 721. | 849.93 | {393., 1047.} | 34.9241 | 0.164763 | Reff | 1.75684 | 1.53941 | {0.905329, 2.51792} | 30.5075 | ar | 9147. | 9333.85 | {9246., 9393.} | 2.05747 |
| 25 | R0 | {2.53191, 10.9214} | {2.71403, 18.1607} | {{2.48143, 15.7026}, {2.85956, 19.8999}} | {8.49776, 66.2856} | inf | 473. | 493.819 | {90., 912.} | 60.4504 | 0.330927 | newly | 441. | 252.25 | {52., 482.} | 49.9937 | 0.429106 | Reff | 2.32379 | 2.48114 | {2.20701, 2.79408} | 10.0028 | ar | 9009. | 9134.14 | {8885., 9335.} | 2.37016 |
| 26 | R0 | {1.89155, 6.76944} | {1.96027, 6.25958} | {{1.56331, 3.72349}, {2.35943, 8.55113}} | {15.3836, 25.7} | inf | 395. | 707.596 | {166., 1245.} | 107.103 | 0.340673 | newly | 577. | 937.365 | {297., 1543.} | 88.4382 | 0.298327 | Reff | 1.71999 | 1.74463 | {1.19343, 2.14539} | 20.5668 | ar | 7348. | 7969. | {6452., 8958.} | 13.6296 |
| 27 | R0 | {2.63149, 15.6979} | {2.57895, 13.4486} | {{2.34121, 11.1899}, {2.76203, 15.3124}} | {6.25433, 14.3288} | inf | 797. | 746.754 | {192., 1426.} | 47.477 | 0.283969 | newly | 482. | 519.652 | {148., 954.} | 49.2513 | 0.258874 | Reff | 2.28124 | 2.22879 | {1.90153, 2.6057} | 10.6701 | ar | 9104. | 8984.48 | {8683., 9267.} | 2.3409 |
| 28 | R0 | {2.85141, 12.6992} | {2.61377, 9.28456} | {{2.21381, 7.10188}, {2.98791, 11.317}} | {10.4951, 26.8887} | inf | 622. | 1251.27 | {428., 2168.} | 118.908 | 0.341888 | newly | 530. | 1211.75 | {518., 1760.} | 139.484 | 0.375879 | Reff | 2.57824 | 1.99872 | {1.44363, 2.64669} | 24.1048 | ar | 9317. | 9007.85 | {8442., 9401.} | 3.74781 |
| 29 | R0 | {2.54244, 12.9085} | {2.84395, 19.7786} | {{2.85956, 19.8999}, {2.86095, 19.9929}} | {12.0203, 53.2212} | inf | 820. | 704.955 | {97., 1479.} | 58.3795 | 0.398869 | newly | 612. | 314.742 | {55., 641.} | 53.4735 | 0.488687 | Reff | 2.19184 | 2.50624 | {2.07661, 2.81038} | 16.82 | ar | 9034. | 9283.21 | {9231., 9357.} | 2.91995 |
| 30 | R0 | {2.10982, 11.7636} | {2.81059, 17.8334} | {{2.5458, 15.1849}, {2.97157, 19.3547}} | {33.2146, 51.5979} | inf | 762. | 1583.7 | {403., 2461.} | 123.78 | 0.367294 | newly | 521. | 714.899 | {302., 1037.} | 58.6358 | 0.248194 | Reff | 1.77373 | 2.00242 | {1.36902, 2.65153} | 22.4647 | ar | 8224. | 9236.9 | {8920., 9414.} | 12.3164 |
| 31 | R0 | {2.76312, 15.5396} | {2.18075, 8.33041} | {{1.84836, 6.13642}, {2.85273, 12.8842}} | {22.2662, 46.3924} | inf | 1053. | 879.156 | {153., 1526.} | 44.2011 | 0.301359 | newly | 607. | 907.234 | {284., 1475.} | 75.3218 | 0.27869 | Reff | 2.27211 | 1.77449 | {1.22583, 2.30843} | 23.3029 | ar | 9220. | 8256. | {7456., 9309.} | 10.7602 |
| 32 | R0 | {2.21727, 15.5439} | {2.4112, 19.5704} | {{2.39393, 19.5287}, {2.42625, 19.7817}} | {8.74652, 25.9043} | inf | 1094. | 1251.15 | {122., 2154.} | 56.8144 | 0.317337 | newly | 577. | 491.083 | {62., 797.} | 38.5124 | 0.292103 | Reff | 1.72703 | 1.78003 | {1.20754, 2.33114} | 20.4352 | ar | 8359. | 8798.37 | {8710., 8879.} | 5.25621 |
| 33 | R0 | {2.72912, 10.8159} | {2.67145, 12.8182} | {{2.45095, 11.4067}, {2.81617, 13.9425}} | {5.44972, 19.8257} | inf | 759. | 697.329 | {238., 1238.} | 46.6196 | 0.265853 | newly | 733. | 525.243 | {202., 944.} | 42.8435 | 0.28753 | Reff | 2.39835 | 2.37342 | {1.9631, 2.63419} | 8.17408 | ar | 9196. | 9140.54 | {8945., 9317.} | 1.46554 |
| 34 | R0 | {2.20928, 10.1308} | {1.82629, 4.42874} | {{1.82289, 4.40955}, {1.82289, 4.40955}} | {17.6405, 56.2844} | inf | 601. | 957.478 | {390., 1217.} | 72.6543 | 0.248922 | newly | 568. | 1548.46 | {865., 1904.} | 173.335 | 0.421451 | Reff | 1.96317 | 1.19004 | {0.792046, 1.66284} | 40.1064 | ar | 8388. | 7380.2 | {7261., 7538.} | 12.3575 |
| 35 | R0 | {2.10103, 15.2673} | {2.51728, 14.0402} | {{2.15962, 10.7247}, {2.85582, 17.2484}} | {20.6541, 12.6122} | inf | 1074. | 1930.31 | {1195., 2470.} | 85.4908 | 0.267529 | newly | 563. | 986.571 | {768., 1165.} | 79.2641 | 0.255992 | Reff | 1.64364 | 1.22917 | {0.666901, 1.92726} | 34.6212 | ar | 8264. | 8874.4 | {8179., 9292.} | 8.2696 |
| 36 | R0 | {2.74914, 19.2775} | {2.63058, 19.6127} | {{2.64373, 19.7855}, {2.64373, 19.7855}} | {4.31259, 3.3207} | inf | 1325. | 1259.15 | {228., 2250.} | 46.2873 | 0.303771 | newly | 562. | 516.161 | {112., 872.} | 41.3385 | 0.274965 | Reff | 2.13223 | 2.02249 | {1.43211, 2.53269} | 15.8815 | ar | 9301. | 9081.02 | {9020., 9136.} | 2.36516 |
| 37 | R0 | {2.78161, 16.0136} | {2.85122, 16.8954} | {{2.59564, 14.6975}, {2.98558, 18.1089}} | {4.3892, 7.92178} | inf | 1098. | 1039.32 | {286., 2082.} | 50.6179 | 0.308011 | newly | 605. | 535.985 | {161., 977.} | 43.2295 | 0.288921 | Reff | 2.28481 | 2.3404 | {1.72837, 2.80574} | 14.2032 | ar | 9240. | 9273.91 | {9057., 9405.} | 1.2694 |
| 38 | R0 | {2.4454, 8.33501} | {2.67687, 12.8807} | {{2.32678, 10.0266}, {2.95376, 15.278}} | {11.1432, 55.1374} | inf | 351. | 352.456 | {78., 727.} | 56.2133 | 0.327402 | newly | 491. | 285.162 | {52., 590.} | 50.9255 | 0.419324 | Reff | 2.30772 | 2.54665 | {2.25418, 2.82858} | 12.0214 | ar | 8853. | 8997.75 | {8694., 9394.} | 5.15727 |
| 39 | R0 | {2.9154, 12.3861} | {2.75052, 11.4813} | {{2.48617, 9.80052}, {2.8186, 12.1249}} | {5.97651, 7.91647} | inf | 746. | 647.296 | {115., 1314.} | 50.7231 | 0.362727 | newly | 645. | 585.831 | {109., 1085.} | 47.6973 | 0.328035 | Reff | 2.57605 | 2.44796 | {2.10015, 2.77323} | 9.43713 | ar | 9371. | 9185.54 | {8857., 9345.} | 2.0116 |
| 40 | R0 | {2.40923, 15.1963} | {1.96788, 8.28423} | {{1.78724, 6.63253}, {2.16209, 9.852}} | {18.319, 45.4852} | inf | 1012. | 997.482 | {551., 1523.} | 33.1469 | 0.175794 | newly | 547. | 941.679 | {595., 1275.} | 80.1711 | 0.260394 | Reff | 1.91847 | 1.45681 | {1.05447, 1.86437} | 24.8878 | ar | 8735. | 7843.61 | {7210., 8656.} | 10.2253 |
| 41 | R0 | {2.63137, 15.238} | {2.8975, 18.226} | {{2.62879, 15.3681}, {2.99088, 18.9406}} | {11.0992, 20.6834} | inf | 1144. | 1688.08 | {635., 2793.} | 71.0357 | 0.263778 | newly | 680. | 729.581 | {328., 1074.} | 38.13 | 0.194909 | Reff | 2.09194 | 2.01484 | {1.2283, 2.658} | 20.5793 | ar | 9150. | 9341.38 | {9271., 9437.} | 2.35209 |
| 42 | R0 | {2.48769, 19.4593} | {2.48906, 19.2578} | {{2.48947, 19.255}, {2.48947, 19.255}} | {0.13435, 1.06364} | inf | 1677. | 1566.45 | {517., 2359.} | 34.4971 | 0.238621 | newly | 673. | 611.403 | {237., 849.} | 28.1815 | 0.19989 | Reff | 1.67844 | 1.65127 | {1.06699, 2.25646} | 22.6007 | ar | 8932. | 8910.47 | {8835., 8981.} | 0.502904 |
| 43 | R0 | {2.61199, 10.214} | {2.65979, 11.2914} | {{2.28593, 9.04943}, {2.93502, 13.2975}} | {7.90142, 15.9003} | inf | 259. | 273.588 | {53., 590.} | 60.5383 | 0.322583 | newly | 315. | 282.456 | {61., 596.} | 53.0486 | 0.324725 | Reff | 2.50594 | 2.52406 | {2.17852, 2.83709} | 8.3066 | ar | 9061. | 9047.68 | {8596., 9376.} | 2.74966 |
| 44 | R0 | {2.50994, 18.3382} | {2.45103, 19.1198} | {{2.43839, 19.0531}, {2.48947, 19.255}} | {2.34716, 4.2622} | inf | 1225. | 1248.46 | {323., 2123.} | 52.9239 | 0.331875 | newly | 597. | 506.381 | {135., 808.} | 40.0495 | 0.301403 | Reff | 1.9314 | 1.83089 | {1.30625, 2.31988} | 18.556 | ar | 8827. | 8871.6 | {8790., 8948.} | 0.708504 |
| 45 | R0 | {2.44283, 16.8338} | {2.43728, 13.3301} | {{2.14747, 10.7214}, {2.74988, 16.4329}} | {7.30764, 21.306} | inf | 1233. | 1297.99 | {494., 2220.} | 40.5303 | 0.190364 | newly | 606. | 798.333 | {360., 1157.} | 50.3396 | 0.189439 | Reff | 1.87707 | 1.747 | {1.09458, 2.29912} | 19.1064 | ar | 8878. | 8736.3 | {7888., 9226.} | 3.40542 |
| 46 | R0 | {2.51815, 18.1462} | {2.72445, 19.2586} | {{2.59755, 18.2464}, {2.76795, 19.6041}} | {8.19247, 6.55916} | inf | 1241. | 2146.63 | {1558., 2630.} | 74.2863 | 0.234424 | newly | 572. | 752.467 | {528., 954.} | 35.7366 | 0.128525 | Reff | 1.943 | 0.82423 | {0.432631, 1.49718} | 58.6872 | ar | 8978. | 9171.96 | {8991., 9270.} | 2.23197 |
| 47 | R0 | {2.88885, 16.8987} | {2.80944, 19.4304} | {{2.56757, 16.884}, {2.86095, 19.9929}} | {2.74869, 15.8409} | inf | 890. | 790.73 | {123., 1828.} | 53.3226 | 0.38176 | newly | 505. | 352.365 | {67., 726.} | 46.9464 | 0.394784 | Reff | 2.47083 | 2.44775 | {1.96824, 2.76948} | 9.26696 | ar | 9337. | 9267.79 | {9077., 9354.} | 0.842866 |
| 48 | R0 | {2.58709, 13.8786} | {2.43346, 10.8935} | {{2.10405, 8.58066}, {2.7943, 13.5962}} | {9.18162, 21.5725} | inf | 706. | 777.97 | {218., 1436.} | 51.8118 | 0.255876 | newly | 483. | 666.567 | {214., 1151.} | 65.3657 | 0.262991 | Reff | 2.27612 | 2.10393 | {1.5995, 2.57941} | 13.3536 | ar | 9146. | 8784.16 | {8156., 9258.} | 4.44383 |
| 49 | R0 | {2.42959, 10.7132} | {2.55392, 10.9868} | {{2.12684, 8.23415}, {2.91762, 13.2975}} | {12.8538, 18.1612} | inf | 413. | 583.913 | {109., 1108.} | 77.5696 | 0.315657 | newly | 380. | 543.464 | {117., 933.} | 77.807 | 0.314191 | Reff | 2.25199 | 2.31635 | {1.79963, 2.74137} | 13.587 | ar | 8863. | 8912.45 | {8277., 9360.} | 4.94516 |
| 50 | R0 | {2.60513, 13.2796} | {2.61599, 14.985} | {{2.2885, 12.1239}, {2.96797, 18.1089}} | {7.9135, 17.4201} | inf | 466. | 594.813 | {146., 1076.} | 67.5966 | 0.287828 | newly | 376. | 375.719 | {110., 660.} | 49.8753 | 0.269194 | Reff | 2.3863 | 2.33423 | {1.93437, 2.75788} | 9.93637 | ar | 9095. | 8993.11 | {8608., 9380.} | 2.84789 |
